# Linking Flaxseed Intake to Lipid and Metabolic Health: Associations with Metabolic Dysfunction Indices and LDL-Subfractions in Slovak Menopausal Women

**DOI:** 10.3390/metabo16100739

**Published:** 2026-10-01

**Authors:** Petra Lenártová, Martina Gažarová, Piotr Ponichtera, Katarína Ražná, Janka Nôžková

**Affiliations:** 1 Institute of Nutrition and Genomics, Faculty of Agrobiology and Food Resources, Slovak University of Agriculture in Nitra, Trieda Andreja Hlinku 2, 949 76 Nitra, Slovakia; petra.lenartova@uniag.sk; 2 Department of Agronomy, Modern Technologies and Informatics, International Academy of Applied Sciences in Łomża, Studencka 19, 18-402 Łomża, Poland; piotr.ponichtera@wsa.edu.pl; 3 Institute of Plant and Environmental Sciences, Faculty of Agrobiology and Food Resources, Slovak University of Agriculture in Nitra, Trieda Andreja Hlinku 2, 949 76 Nitra, Slovakia; katarina.razna@uniag.sk (K.R.); janka.nozkova@uniag.sk (J.N.)

**Keywords:** flaxseed, menopausal women, lipid profile, lipoprotein subfractions, metabolic indices

## Abstract

**Background/Objectives:** The aim of the present study was to investigate the effects of flaxseed consumption on selected metabolic health indicators in menopausal women following an eight-week dietary intervention. **Methods:** The study cohort comprised 34 menopausal women aged 45–55 years who were voluntarily recruited from the general population. The intervention lasted for eight weeks, during which participants received 40 g of ground flaxseed daily. Biochemical blood parameters were measured using a BioLis 24i Premium automatic analyser. Body composition was assessed using an InBody 720 device. Lipoprotein subfraction analysis was performed using the LipoPrint^®^ LDL System. Metabolic indices were calculated according to established equations. **Results:** Overall, the present findings suggest that the most pronounced changes following flaxseed supplementation occurred in selected lipid parameters, particularly LDL-C, total cholesterol, VLDL, IDL-A, and IDL-B. In contrast, HDL-C, TG, glucose, liver enzymes after multiple-testing correction, anthropometric measurements, and the investigated surrogate indices of adiposity and metabolic dysfunction did not show statistically significant changes. The calculated metabolic indices (C-I, BRI, HSI, LAP, TyG-I, TyG-BMI, TyG-WC, TyG-WHtR) suggest preferential visceral fat deposition and an alteration in adipose tissue distribution. **Conclusions:** Flaxseed supplementation produced a heterogeneous lipid response, with decreased LDL-C but increased total cholesterol, VLDL, and IDL. No significant changes were observed in other lipid, glycemic, anthropometric, or calculated metabolic parameters after correction for multiple comparisons. These findings do not support a uniformly favorable cardiometabolic effect and warrant further investigation.

## 1. Introduction

Chronic non-communicable diseases (NCDs), particularly cardiovascular disease (CVD) and type 2 diabetes mellitus (T2DM), are major causes of morbidity and mortality worldwide. Their increasing prevalence is associated with unhealthy dietary habits, physical inactivity, industrialization, and population ageing [1,2,3].

Metabolic syndrome (MetS) is characterized by a cluster of metabolic abnormalities, including insulin resistance, central obesity, atherogenic dyslipidaemia, and hypertension [4]. Abdominal obesity plays a key role in the development of insulin resistance and associated lipid disturbances, including increased triacylglycerol synthesis and the accumulation of atherogenic VLDL particles [5,6]. Dyslipidaemia is a major modifiable risk factor for CVD and is also associated with T2DM, metabolic dysfunction-associated steatotic liver disease (MASLD), and other metabolic disorders [7,8]. LDL particles are heterogeneous, and small dense LDL (sdLDL) particles have been associated with increased cardiovascular risk. Their detailed assessment may therefore provide additional information beyond conventional lipid profiling [9,10].

The prevalence of MetS has increased considerably in recent decades, with estimates ranging from 12.5% to 32% worldwide [11,12]. Although extensively studied, its complex pathophysiology remains incompletely understood [3]. Consequently, simple metabolic and anthropometric indices have gained increasing attention as practical tools for assessing metabolic risk. Among these, the triglyceride–glucose (TyG) index is widely used as a surrogate marker of insulin resistance. Its combination with anthropometric measures, particularly body mass index (BMI) and waist circumference (WC), resulting in the TyG-BMI and TyG-WC indices, may improve the assessment of metabolic dysfunction and adiposity [13,14,15]. TyG-BMI has also been associated with hepatic steatosis, suggesting its potential utility as an indicator of metabolic dysfunction [16,17].

Lifestyle modification remains the cornerstone of the prevention and management of MetS, with dietary interventions and increased physical activity representing key components of a comprehensive lifestyle-based approach [18,19,20,21,22]. Within this context, flaxseed (*Linum usitatissimum* L.) has attracted considerable interest as a nutrient-dense functional food because of its high content of biologically active compounds, including the omega-3 polyunsaturated fatty acid α-linolenic acid (ALA), lignans, dietary fibre, proteins, and bioactive peptides [23,24,25]. Evidence from clinical and experimental studies suggests that flaxseed supplementation may favourably influence several cardiometabolic risk factors, including body composition, glycaemic regulation, lipid profiles, blood pressure, inflammation, and oxidative stress [19,26,27]. However, the magnitude and consistency of these effects may vary depending on the dose, duration of supplementation, form of flaxseed, and characteristics of the study population [23,24,25].

Among the bioactive constituents of flaxseed, ALA is of particular interest. It accounts for approximately 50–60% of the fatty acids in flaxseed oil and 20–25% of the fatty acids in whole flaxseed, making flaxseed one of the richest plant-based dietary sources of this essential omega-3 fatty acid [25,26,27,28]. Although ALA can be metabolically converted to the long-chain omega-3 fatty acids eicosapentaenoic acid (EPA) and docosahexaenoic acid (DHA), this conversion is limited, particularly in the case of DHA [29]. Therefore, the potential cardiometabolic effects of flaxseed cannot be attributed solely to its conversion to EPA and DHA, as ALA may also exert biological effects through other metabolic pathways [29].

Flaxseed is also one of the richest dietary sources of plant lignans, with secoisolariciresinol diglucoside (SDG) being the predominant lignan. Following ingestion, SDG is metabolized by the gut microbiota to the enterolignans enterodiol and enterolactone, which may contribute to the biological activity of flaxseed through antioxidant, estrogenic, and potentially cardioprotective mechanisms [30,31]. Flaxseed lignans may also influence estrogen metabolism. In postmenopausal women, flaxseed consumption has been associated with increased levels of enterolignans, 2-hydroxyestrone, and the 2:16α-hydroxyestrone ratio [32]. Moreover, flaxseed supplementation has been associated with improvements in menopausal symptoms, particularly reductions in hot-flash severity and overall symptom burden, without significant effects on the endometrium or vaginal epithelium [31,33,34]. These effects were accompanied by improvements in quality of life [33].

The substantial dietary fibre content of flaxseed may represent another important contributor to its metabolic effects. Flaxseed contains both soluble and insoluble fibre, with insoluble fibre generally predominating. The fibre fraction of flaxseed may support gastrointestinal function and contribute to favourable effects on lipid metabolism and glycaemic regulation [35]. In addition, flaxseed provides proteins and bioactive peptides that have demonstrated antioxidant and antihypertensive activities, primarily in experimental studies [29].

Flaxseed also contains cyanogenic glycosides, including linustatin and neolinustatin, which may release hydrogen cyanide. However, available evidence suggests that their presence does not preclude the consumption of flaxseed as a food when consumed appropriately, although safety considerations remain relevant with excessive intake [36]. Mechanical processing, such as grinding, may effectively reduce the levels or activity of cyanogenic glycosides (CGs) [37].

Thus, the potential health benefits of flaxseed should be considered in relation to its overall composition, dose, and form of consumption. However, limited evidence is available regarding the combined effects of flaxseed consumption on anthropometric parameters, metabolic indices, and detailed lipoprotein subfractions. Therefore, this study aimed to investigate the effects of an eight-week dietary intervention with flaxseed on selected metabolic health indicators in menopausal women.

## 2. Materials and Methods

### 2.1. Study Participants

The study cohort comprised 34 menopausal women aged 45–55 years who were voluntarily recruited from the general population. Participant eligibility was determined according to predefined inclusion and exclusion criteria. The inclusion criteria were female sex, age between 45 and 55 years, menopausal status (defined as 12 consecutive months of amenorrhea in the absence of another apparent cause), good general health (defined as the absence of diagnosed acute or chronic conditions that could affect the study outcomes), no known food allergies or intolerances, and no current pharmacological treatment that could influence the study outcomes. Participants were also required to maintain their habitual dietary intake, physical activity, and overall lifestyle throughout the study period. The exclusion criteria were serious acute or chronic medical conditions, ongoing medical treatment, adherence to a physician-prescribed special diet, previous endoprosthesis implantation, or the presence of a cardiac pacemaker.

Participant recruitment was conducted over a one-month period. A total of 40 individuals expressed interest in participating and underwent eligibility screening. Following the screening process, 35 participants met all eligibility criteria and were included in the study. Before the intervention began, one participant withdrew for personal reasons. Thus, 34 participants commenced the study and were included in the subsequent follow-up.

### 2.2. Flaxseed Intervention

The intervention lasted 8 weeks, during which participants consumed 40 g of freshly ground flaxseed daily. The 40-g daily portion of flaxseed was added to the participants’ habitual diet and did not replace any specific food or meal. The flaxseed was cultivated and supplied by AGRITEC Co., Ltd. (Šumperk, Czech Republic). Individual daily portions were packed in light-impermeable, food-grade resealable bags to minimize oxidation and preserve product stability. Freshly ground flaxseed was prepared and distributed to participants weekly. Participants were instructed to store the packages under refrigerated conditions until consumption, as previously described by Lenártová et al. [38]. Adherence to the intervention was monitored throughout the 8-week intervention period. Participants received individually packaged daily portions of 40 g of freshly ground flaxseed weekly. At each weekly visit, participants returned the packages from the preceding week and received the portions for the following week. Adherence was assessed by reviewing the returned packages and through a brief face-to-face interview. Participants were asked whether they had consumed the entire prescribed daily amount and whether they had experienced any self-reported changes, including gastrointestinal discomfort or other adverse effects, as well as any perceived beneficial changes during the preceding week. Any reported symptoms or changes were recorded and monitored throughout the intervention period. No intervention-related adverse events were reported during the study period.

The nutritional composition of the flaxseed was determined prior to the intervention using standardized analytical procedures. Secoisolariciresinol (SECO) content was quantified following Soxhlet extraction of lipids from homogenized flaxseed samples. Following acid hydrolysis of secoisolariciresinol diglucoside (SDG) and other lignan glycosides, the resulting aglycones were extracted with organic solvents and analyzed as derivatized SECO by gas chromatography with flame ionization detection (GC-FID), using nordihydroguaiaretic acid as the internal standard. The fatty acid profile was determined according to the ČSN ISO 5508 standard for the analysis of fatty acid methyl esters by gas chromatography, using Clarity Chromatography software (version 9.1.01.013; DataApex Co., Ltd., Prague, Czech Republic) [39]. The nutritional characteristics of the intervention flaxseed are summarized in Table 1.

### 2.3. Study Design and Methods

The present study investigated the effects of an eight-week flaxseed dietary intervention on selected metabolic health indicators in 34 women from the flaxseed intervention cohort previously described by Lenártová et al. (2024) [38]. Anthropometric measurements and fasting blood samples were obtained at baseline and after the intervention. The current analyses comprised changes in body composition, serum biochemical parameters, lipoprotein subfraction profiles, and selected calculated metabolic dysfunction indices. Specifically, cholesterol subfractions, alanine aminotransferase (ALT), aspartate aminotransferase (AST), fasting blood glucose (FBG), and derived metabolic indices were newly analyzed. Total cholesterol (T-C), LDL-C, HDL-C, and triglyceride (TG) concentrations, although previously reported for this cohort, were included here because they were required for the calculation and interpretation of cholesterol subfractions and derived indices. High-sensitivity C-reactive protein (hs-CRP) was measured at baseline and after the 8-week intervention. However, hs-CRP was not considered an appropriate marker for assessing the inflammatory response within the methodological framework of the present study. The aim of the present analysis was to characterize changes within the studied group rather than to compare changes between intervention and control groups. Therefore, the control group from the previous study by Lenártová et al. (2024) [38] was not included.

#### 2.3.1. Blood Collection and Biochemical Analysis

Blood samples and anthropometric measurements were obtained at baseline and after the 8-week intervention. Venous blood was collected after an overnight fast (10–12 h) into 7.5 mL serum-separation tubes containing separation gel. Following centrifugation at 3000 rpm for 10 min at 4 °C, serum was separated and used for biochemical analyses. Serum biochemical parameters were analysed using a BioLis 24i Premium automated clinical chemistry analyser (Tokyo Boeki Medical System Co., Ltd., Tokyo, Japan), based on endpoint photometric (including turbidimetric), kinetic, and homogeneous immunoassay methods, according to the manufacturer’s instructions. The following biochemical parameters were analysed: total cholesterol (T-C), low-density lipoprotein cholesterol (LDL-C), high-density lipoprotein cholesterol (HDL-C), triacylglycerols (TG), fasting blood glucose (FBG), alanine aminotransferase (ALT), aspartate aminotransferase (AST). The laboratory reference ranges were as follows: T-C < 5.00 mmol/L, LDL-C 1.20–3.00 mmol/L, HDL-C 1.20–2.70 mmol/L, TG 0.45–1.70 mmol/L, FBG 3.9–5.6 mmol/L, ALT 0.17–0.66 µkat/L, and AST 0.16–0.63 µkat/L.

#### 2.3.2. Analysis of Cholesterol Subfractions

Lipoprotein subfraction analysis was performed using the LipoPrint^®^ LDL System (Quantimetrix Corporation, Redondo Beach, CA, USA), a polyacrylamide gel (PAG) electrophoresis method for lipoprotein separation that enables the identification and quantification of up to 12 lipoprotein fractions and subfractions in a single electrophoretic run. The LipoPrint^®^ LDL System was used to determine cholesterol distribution across lipoprotein fractions and subfractions, ranging from very-low-density lipoproteins (VLDL) to high-density lipoproteins (HDL), in fasting serum samples with a total cholesterol concentration of ≥100 mg/dL. The system enables the identification and quantification of intermediate-density lipoprotein subfractions (IDL-C and IDL-B), as well as small, dense LDL particles (LDL3–LDL7), which are considered highly atherogenic lipoprotein subfractions.

#### 2.3.3. Anthropometric Measurements

Anthropometric measurements were performed using the InBody 720 body composition analyser (Biospace Co., Ltd., Seoul, Republic of Korea), which is based on the principle of bioelectrical impedance analysis (BIA). The analyser provides comprehensive body composition assessment, including fat mass (FM), fat-free mass (FFM), visceral fat area (VFA), percent body fat (PBF), skeletal muscle mass (SMM), total body water (TBW), intracellular water (ICW), and extracellular water (ECW). Participants were assessed under standardized conditions following an overnight fast or at least 2 h after the last meal and after bladder emptying. Before the assessment, participants were instructed to avoid excessive fluid intake, vigorous physical activity, showering, and sauna use, as these factors may temporarily affect body composition measurements. All measurements were performed at a room temperature of 20–25 °C. Body composition data were processed using Lookin’Body 3.0 software (Biospace Co., Ltd., Seoul, Republic of Korea). To assess metabolic risk, the following anthropometric parameters were evaluated: visceral fat area (VFA, cm^2^), percent body fat (PBF, %), and body mass index (BMI, kg/m^2^). Body height and body weight were measured using a TANITA WB-380H stadiometer with an integrated scale (TANITA Corporation, Tokyo, Japan). Participants were measured barefoot and wearing light clothing while standing in an upright position. Body mass index (BMI) was calculated as body weight (kg) divided by the square of body height (m^2^).

#### 2.3.4. Calculation of Metabolic Dysfunction Indices

To evaluate the metabolic risk profile of the study population, a panel of validated surrogate indices reflecting different aspects of metabolic dysfunction was calculated. The equations used to calculate the metabolic dysfunction indices are presented in Table 2. These included the Conicity Index (C-I), a marker of abdominal adiposity; the Body Roundness Index (BRI), which estimates body fat distribution and visceral adiposity based on waist circumference and height; the Hepatic Steatosis Index (HSI), calculated to estimate hepatic steatosis; and the Lipid Accumulation Product (LAP), which served as an indicator of visceral lipid accumulation. In addition, the Triglyceride–Glucose index (TyG-I), together with its anthropometric derivatives (TyG-BMI, TyG-WC, and TyG-WHtR), was included, as these indices have been proposed as reliable surrogate markers of insulin resistance. The combined assessment of these indices provided a comprehensive characterization of body fat distribution, abdominal obesity, visceral adiposity, metabolic dysfunction, metabolic dysfunction-associated steatotic liver disease (MASLD), and insulin resistance (IR), all of which are closely associated with increased cardiometabolic risk.

### 2.4. Statistical Analysis

Data processing was performed using Microsoft Office Excel 2016 (Microsoft Corporation, Redmond, WA, USA) and XLSTAT 2019 (Addinsoft, New York, NY, USA). Statistical analyses were performed using STATISTICA 13 (TIBCO Software, Inc., Palo Alto, CA, USA) and MedCalc Statistical Software version 22.021 (MedCalc Software Ltd., Ostend, Belgium; 2024). Continuous variables are presented as medians with interquartile ranges (IQRs). Statistical analyses were based on 34 paired observations, with measurements obtained before and after the intervention from the same participants. The distribution of the within-subject differences between pre- and post-intervention measurements was assessed using the Shapiro–Wilk test. As the differences deviated significantly from normality (*p* < 0.05), non-parametric methods were used for paired comparisons. Pre- and post-intervention values were therefore compared using the Wilcoxon signed-rank test. For each outcome variable, the Wilcoxon test statistic (Z), two-sided *p*-value, and rank-biserial correlation (r_r_b) as a measure of effect size were reported. Ninety-five percent confidence intervals (95% CIs) for the rank-biserial correlation were estimated using a bootstrap procedure based on the paired observations. To account for multiple comparisons across outcome variables, *p*-values were adjusted using the Holm method. Statistical significance was defined as a two-sided Holm-adjusted *p*-value < 0.05.

Associations between the variables under study were assessed using Spearman’s rank correlation coefficient (ρ), which was used to quantify the strength and direction of monotonic relationships. No adjustment for multiple comparisons was applied. Therefore, the reported *p*-values represent nominal significance levels. Correlation analyses were performed using MedCalc Statistical Software version 22.021 (MedCalc Software Ltd., Ostend, Belgium; 2024).

### 2.5. Ethical Considerations

The study was conducted in accordance with the principles of the Declaration of Helsinki and was approved by the Ethics Committee of St. Zoerard Zobor Specialized Hospital, Kláštorská 131, 949 01 Nitra, Slovak Republic (Protocol No. 5/071220/2020; approval date: 10 December 2020). Written informed consent was obtained from all participants prior to their enrolment in the study.

## 3. Results

Thirty-four women were included in the analysis, with a median age of 48.5 years (IQR: 47.0–52.5 years; range: 45–55 years). At baseline, the median BMI was 24.35 kg/m^2^ (IQR: 22.50–30.60), while WHR (0.93), WHtR (0.54), WC (89.75 cm), and VFA (96.55 cm^2^) indicated predominantly central adiposity and increased cardiometabolic risk. The baseline anthropometric and biochemical characteristics of the study participants are presented in Table 3.

### 3.1. Assessment of Biochemical Parameters in Relation to Flaxseed Intervention

The effects of flaxseed intervention on selected biochemical parameters were assessed by comparing their values before and after the intervention (Table 4).

Total cholesterol (T-C) increased significantly from a median of 5.51 mmol/L (IQR, 4.98–6.27) before the intervention to 6.18 mmol/L (IQR, 5.58–6.78) after the intervention (Holm-adjusted *p* < 0.001).

In contrast, LDL-C decreased significantly from 3.34 mmol/L (IQR, 2.74–3.89) to 2.89 mmol/L (IQR, 2.28–3.18; Holm-adjusted *p* < 0.001). Similarly, AST decreased significantly from 0.29 µkat/L (IQR, 0.25–0.36) to 0.28 µkat/L (IQR, 0.24–0.34; Holm-adjusted *p* = 0.023). For HDL-C and TG, trends toward slight increases were observed, while FBG and ALT showed trends toward decreases. However, these changes were not statistically significant after Holm adjustment.

### 3.2. Assessment of LDL Subfractions in Relation to Flaxseed Intervention

Changes in selected lipoprotein subfractions following the flaxseed intervention are presented in Table 5. Statistically significant increases were observed in VLDL, IDL-B, and IDL-A concentrations after the intervention (Holm-adjusted *p* = 0.005, *p* = 0.010, and *p* < 0.001, respectively).

IDL-C, LDL-1, and HDL-C showed trends toward increases, whereas LDL-2 and LDL-3–7 showed trends toward decreases. A slight increasing trend was also observed for mean LDL particle size (m-LDL size). None of these latter changes were statistically significant after Holm adjustment.

The observed trends toward a decrease in LDL-3–7 subfractions and an increase in mean LDL particle size may be compatible with a more favourable lipoprotein profile.

### 3.3. Assessment of Anthropometric Parameters and Metabolic Indices in Relation to Flaxseed Intervention

Changes in anthropometric parameters and selected metabolic indices following the flaxseed intervention are presented in Table 6. No statistically significant changes were observed in any of the assessed parameters after Holm adjustment. Overall, BMI, WHR, WHtR, and HC showed slight increasing trends, whereas WC remained largely unchanged. VFA showed a trend toward a decrease, while C-I, BRI, HSI, and LAP showed slight increasing trends. Among the TyG-related indices, TyG-I showed a slight increasing trend, whereas TyG-BMI and TyG-WC showed slight decreasing trends; TyG-WHtR remained largely unchanged.

### 3.4. Correlations Between Monitored Parameters and Indices in Relation to Flaxseed Intervention

Table 7 presents the correlation relationships between selected anthropometric parameters (BMI; WC; VFA; WHtR), metabolic indices (LAP, HSI, TyG-I; TyG-BMI; TyG-WC; TyG-WHtR), biochemical parameters (FBG; ALT; TG) and lipoprotein subfractions (T-C; LDL-C; HDL-C; IDL-C; LDL-3–7; m-LDL-size). To facilitate the distinction of mathematically derived correlations, the respective parameters are indicated as “mathematical corr.”.

A strong correlation was confirmed between TyG-I and BMI; LAP; HSI; TG (mathematical corr.); and IDL-C. In the case of TyG-BMI, a strong correlation was observed with BMI (mathematical corr.); WC; VFA; WHtR; LAP; HSI (mathematical corr.); TG (mathematical corr.); and IDL-C.

A strong correlation was confirmed between TyG-WC and BMI; WC (mathematical corr.); VFA; WHtR (mathematical corr.); LAP (mathematical corr.); HSI; and IDL-C. In the case of TyG-WHtR, a strong correlation was observed with BMI; WC (mathematical corr.); VFA; WHtR (mathematical corr.); LAP (mathematical corr.); HSI; TG (mathematical corr.); and IDL-C.

## 4. Discussion

The aim of the present study was to investigate the effects of flaxseed consumption on selected metabolic health indicators in menopausal women following an eight-week intervention. The study cohort comprised menopausal women from the general population who had no history of serious medical conditions. Evidence from multiple clinical studies indicates that menopause represents a critical stage in the onset and progression of cardiovascular disease, owing to several underlying factors [41,42,43]. Premenopausal women are generally protected against the clinical manifestations of cardiovascular disease (CVD). Following menopause, however, the decline in estrogen levels is associated with an increased risk of cardiovascular complications. Although the risk of CVD is lower in premenopausal women than in age-matched men, this protective effect diminishes after menopause, resulting in a substantial increase in cardiovascular risk [44].

During the menopausal transition, women experience significant changes in lipid metabolism, including increases in total cholesterol, LDL cholesterol, and triglycerides, as well as adverse alterations in HDL cholesterol and Lp(a) levels. These changes, influenced by declining estrogen levels and hormonal shifts, contribute to an elevated risk of atherosclerotic cardiovascular disease in postmenopausal women [45,46].

Several studies have demonstrated that postmenopausal women have significantly higher levels of triglycerides, total cholesterol, LDL cholesterol, and the total cholesterol-to-HDL cholesterol ratio than premenopausal women. However, no significant difference in HDL cholesterol levels was observed between premenopausal and postmenopausal women [47,48,49].

Although dyslipidaemia is a well-established risk factor for cardiovascular disease (CVD), many individuals who have experienced acute vascular events present with LDL cholesterol levels within the normal range. Conversely, elevated LDL cholesterol concentrations have also been reported in individuals without clinical or laboratory evidence of CVD [50,51]. The findings of our study indicate that the changes observed after supplementation were predominantly evident in selected lipid parameters, whereas most anthropometric and surrogate metabolic indices remained statistically unchanged.

TG levels remained relatively stable and, despite a slight, non-significant increase, remained within the normal range. A statistically significant increase in total cholesterol and a concurrent decrease in LDL cholesterol was observed. HDL cholesterol showed a slight increasing trend, although the change did not reach statistical significance.

The importance of analysing cholesterol subfractions has increased in recent years. Several studies have demonstrated that an atherogenic lipoprotein profile, characterised by increased concentrations of very-low-density lipoproteins (VLDL), intermediate-density lipoproteins (IDL1–3), small HDL particles, and, in particular, small dense LDL subfractions (LDL-3–7), is associated with an increased risk of atherogenesis and cardiovascular disease (CVD). In contrast, an anti-atherogenic lipoprotein profile, characterised by a higher proportion of LDL1–2 and large and medium-sized HDL particles, together with only trace amounts of LDL3–7 subfractions, has been associated with greater protection against cardiovascular disease [50,52].

In our study, the lipid response was not uniformly favourable, as significant decreases in LDL-C occurred alongside increases in total cholesterol, VLDL, IDL-B, and IDL-A. These findings may indicate a mixed lipid response. A significant decrease in LDL-C was observed following flaxseed supplementation, with median values decreasing from 3.34 to 2.89 mmol/L (Holm-adjusted *p* < 0.001). In contrast, total cholesterol increased significantly from 5.51 to 6.18 mmol/L (Holm-adjusted *p* < 0.001). HDL-C showed a small increase, whereas TG also increased slightly; however, neither change reached statistical significance after Holm adjustment. These findings suggest that different lipid fractions may have responded differently during the intervention period.

The analysis of cholesterol subfractions further supports the heterogeneous nature of the lipid response. Significant increases were observed in VLDL, IDL-B, and IDL-A, while IDL-C, LDL-1, LDL-2, LDL-3–7, and mean LDL particle size did not change significantly after Holm adjustment. Thus, although the reduction in LDL-C may be considered potentially favourable from a lipid-related perspective, its interpretation should consider the concurrent increases in total cholesterol and several triglyceride-rich lipoprotein fractions.

Duan et al. [53] evaluated 566 patients with acute ischemic stroke (AIS) and demonstrated that, compared with individuals without AIS, these patients exhibited significantly higher concentrations of the LDL-3, LDL-4, and LDL-5 subfractions, accompanied by significantly lower levels of the LDL-1 subfraction. Comparable results were reported by Chang et al. [54]. Likewise, in a cross-sectional study investigating the relationship between LDL subfractions and AIS outcomes, Zitnanova et al. [55] observed significantly reduced serum concentrations of the anti-atherogenic large LDL-1 subfraction in patients with AIS, whereas the concentrations of the more atherogenic LDL-3 and LDL-5 subfractions were significantly increased.

Changes in VLDL and IDL fractions may reflect alterations in lipoprotein metabolism during the intervention, although this remains speculative. Flaxseed provides dietary fat together with fibre and other bioactive components, and its overall nutritional composition could potentially influence lipid transport and metabolism. However, the present study did not directly assess these mechanisms. In addition, although participants were instructed to maintain their habitual diet and physical activity, unmeasured changes in dietary intake or lifestyle cannot be completely excluded.

The biochemical findings should also be considered in the context of the observed changes in glucose and liver enzymes. Fasting blood glucose decreased slightly from a median of 4.77 to 4.63 mmol/L, but this difference was not statistically significant after Holm adjustment. ALT showed a decrease in the unadjusted analysis; however, this change did not remain statistically significant after correction for multiple comparisons (Holm-adjusted *p* = 0.938). AST concentrations were lower after the intervention, decreasing from 0.29 µkat/L (IQR, 0.25–0.36) to 0.28 µkat/L (IQR, 0.24–0.34; Holm-adjusted *p* = 0.023). While this difference reached statistical significance after Holm adjustment, its interpretation should remain cautious given the exploratory design and small sample size (n = 34). The small magnitude of the observed change limits conclusions about its clinical relevance, and the finding should be regarded as preliminary and hypothesis-generating rather than as evidence of a clinically meaningful change in liver enzyme status.

In the study by Matsui et al. [56], both AST and ALT levels were found to increase during the transition to early post menopause. In late post menopause, AST levels remained elevated, whereas ALT levels decreased. Changes in ALT levels and the AST/ALT ratio during the menopausal transition, in association with triglyceride levels, may contribute to the development of metabolic syndrome and metabolic dysfunction-associated steatotic liver disease (MASLD) in women.

Menopause is associated with a significant increase in blood pressure, body mass index (BMI), obesity, and body fat distribution [57].

Changes in fat distribution and body composition that occur during menopause are associated with increased cardiometabolic risk factors, including elevated blood pressure, elevated low-density lipoprotein (LDL) cholesterol levels, and obesity. Changes in visceral fat increase CVD risk through their association with insulin resistance, inflammation, and an adverse lipid profile [58].

In the present cohort, baseline values showed considerable interindividual variability in body mass index (BMI), waist circumference (WC), and visceral fat area (VFA). However, none of the anthropometric variables assessed in the present study, including body mass index (BMI), waist-to-hip ratio (WHR), waist-to-height ratio (WHtR), waist circumference (WC), hip circumference (HC), and visceral fat area (VFA), changed significantly following supplementation. The median visceral fat area decreased slightly from 96.55 to 92.95 cm^2^, but this change was not statistically significant (Holm-adjusted *p* = 1.000). Therefore, the present findings do not provide evidence that the supplementation period was accompanied by a measurable change in body composition or abdominal adiposity. Cardiometabolic risk based on anthropometric variables remained relatively unchanged.

Visceral adiposity is an independent risk factor for cardiovascular disease. Traditional anthropometric measures, such as body mass index (BMI), waist circumference (WC), and waist-to-hip ratio (WHR), have limited accuracy, whereas a newer measure, the Body Roundness Index (BRI), has been proposed as a better predictor of visceral adiposity [59].

To provide a more comprehensive assessment of obesity, insulin resistance risk, and cardiovascular risk, we also evaluated body shape indices, namely the Body Roundness Index (BRI) and the Conicity Index (C-I).

The Body Roundness Index (BRI), which incorporates waist circumference and height, better reflects visceral adiposity and can more precisely capture differences in fat distribution [60]. Incorporating the BRI into clinical risk assessment can improve the identification of high-risk individuals—particularly those with abdominal obesity—whom BMI might fail to detect [61]. The C-I is widely used to assess abdominal visceral adiposity and is recognised as an indicator of body fat distribution, with higher values reflecting greater accumulation of abdominal fat [62]. Compared with BMI, it has been shown to be a more effective anthropometric measure for identifying body fat distribution, particularly central obesity [63]. For example, a cross-sectional study conducted in a Mexican population demonstrated that the C-I was a more accurate predictor of adiposity-based chronic diseases [64]. Likewise, research involving Brazilian women aged 20–59 years indicated that the C-I is a valuable tool for estimating the risk of diabetes, hypertension, and dyslipidaemia [65]. The Body Roundness Index (BRI) and Conicity Index (C-I), which are indirect anthropometric measures proposed as markers of body shape and central adiposity, remained statistically unchanged. Median BRI values changed from 4.14 to 4.23, while C-I values changed from 1.27 to 1.29, with neither difference reaching statistical significance after adjustment for multiple comparisons. The BRI values observed in menopausal women in our study may suggest a slight tendency toward increased body roundness. This phenomenon is consistent with the physiological changes associated with menopause, a period typically characterised by increased visceral fat accumulation. Declining levels of sex hormones contribute to changes in fat distribution, potentially leading to an increased cardiometabolic risk.

Previous studies have frequently associated such changes with deterioration of the lipid profile, particularly reduced HDL cholesterol levels [66,67]. In contrast, our exploratory study revealed a heterogeneous lipid response following flaxseed supplementation. LDL-C decreased significantly, whereas HDL-C and TG concentrations remained essentially unchanged. A numerical trend toward lower LDL-3–7 concentrations and a slight increase in mean LDL particle size was also observed; however, neither change reached statistical significance. Interestingly, total cholesterol increased significantly despite the reduction in LDL-C. This increase coincided with significant increases in VLDL, IDL-A, and IDL-B concentrations, suggesting that the change in total cholesterol may have reflected alterations in cholesterol carried by these other lipoprotein fractions. Overall, the lipid changes observed following flaxseed supplementation were heterogeneous, with significant changes in some lipoprotein fractions but only non-significant trends in others. Given the exploratory nature of the study, these findings should be interpreted cautiously and considered hypothesis-generating rather than as evidence of a causal lipid-modifying effect of flaxseed supplementation.

To further evaluate metabolic alterations, we assessed screening indices associated with the risk of metabolic dysfunction-associated steatotic liver disease (MASLD), namely the Hepatic Steatosis Index (HSI) and the Lipid Accumulation Product (LAP).

Huang et al. [68] state that the Hepatic Steatosis Index (HSI) is a widely recognised indicator for assessing hepatic fat accumulation. In females, a significant positive association with prediabetes was observed when HSI values were below a threshold of 36.48. Above this cut-off, the association weakened and was no longer statistically significant.

The HSI values observed in our study remained essentially unchanged, with median values of 33.16 before and 33.25 after supplementation (Holm-adjusted *p* = 1.000). The present results therefore indicate no detectable change in HSI during the intervention. The HSI values observed in the study participants fell within a borderline or at-risk range. Although these values may be indicative of an increased risk of hepatic steatosis, HSI alone cannot establish the presence or absence of hepatic steatosis or metabolic dysfunction-associated steatotic liver disease (MASLD). Such a metabolic state may be reversible through dietary modifications, increased physical activity, stress reduction, and adequate sleep.

The Lipid Accumulation Product (LAP), another surrogate index related to central lipid accumulation and metabolic risk [69], also showed no statistically significant change. Median LAP increased slightly from 30.40 to 31.11, but the difference did not remain significant after Holm adjustment (Holm-adjusted *p* = 1.000). This result indicates that the supplementation period was not associated with a statistically detectable change in this surrogate marker. Given the indirect nature of LAP, these findings cannot be used to determine whether insulin resistance, metabolic syndrome, or hepatic steatosis was present or absent in individual participants. In addition, the LAP values in menopausal women fell within a range indicative of mild to moderate metabolic risk. Abdominal adiposity (visceral fat), combined with higher concentrations of circulating lipids, may create conditions conducive to the development of insulin resistance. When interpreting HSI and LAP, it is important to consider the hormonal changes associated with menopause, particularly oestrogen deficiency, which has a substantial impact on lipid metabolism. Therefore, we consider the assessment of HSI and LAP to be particularly valuable during the menopausal transition.

Finally, our comprehensive assessment of the nutritional and health status of the menopausal women included indices associated with insulin resistance, namely the Triglyceride–Glucose (TyG) index and its anthropometric derivatives (TyG-BMI, TyG-WC, and TyG-WHtR). The TyG index is widely used as a sensitive marker of insulin resistance (IR) [70], diabetes mellitus [71], and cardiovascular and cerebrovascular events [72]. Elevated TyG index values have also been associated with metabolic syndrome [73]. Furthermore, the TyG index has been recognised as a marker of cardiometabolic risk [74].

Recent studies have shown that TyG-related indices, including the triglyceride–glucose–body mass index (TyG-BMI), triglyceride–glucose–waist-to-height ratio (TyG-WHtR), and triglyceride–glucose–waist circumference (TyG-WC), combine the TyG index with obesity-related anthropometric measures, such as body mass index (BMI), waist-to-height ratio (WHtR), and waist circumference (WC), thereby providing a more accurate assessment of insulin resistance than the TyG index alone [75,76,77].

In the present study, TyG-I, TyG-BMI, TyG-WC, and TyG-WHtR remained statistically unchanged following supplementation. For example, median TyG-I increased from 8.08 to 8.20, whereas TyG-BMI changed from 199.84 to 199.66. TyG-WC and TyG-WHtR also showed only small changes, none of which remained statistically significant after correction for multiple comparisons.

These results therefore do not demonstrate a measurable change in the evaluated surrogate markers of metabolic dysfunction during the intervention. Nevertheless, the observed TyG index values may suggest that the participants had some degree of metabolic risk associated with visceral adiposity and excess body weight. The TyG index values were consistent with preserved insulin sensitivity, although they were close to the established risk threshold. The other TyG-related indices likewise suggested a potential mild metabolic impact of excess body weight and adiposity.

Cui et al. [78] found that TyG and TyG-WHtR were significantly associated with new-onset cardiovascular diseases, and TyG outperformed the modified TyG indices for identifying individuals at risk of incident cardiovascular events.

Several studies [79,80,81,82,83] have reported that TyG-WHtR has the highest predictive accuracy for identifying individuals at risk of developing type 2 diabetes mellitus or metabolic syndrome. In the study by Al Akl et al. [84], the TyG-WHtR index was found to be significantly associated with prediabetes even among healthy adult women with normal body weight. These findings suggest that the TyG-WHtR index may be a more suitable indicator of prediabetes for general clinical use in adults with normal body weight than other obesity- and TyG-related indices. Furthermore, when combined with fasting plasma glucose (FPG) measurements, the TyG-WHtR index may help identify adults at risk of developing prediabetes.

Overall, the present findings suggest that the most pronounced changes following flaxseed supplementation occurred in selected lipid parameters, particularly LDL-C, total cholesterol, VLDL, IDL-A, and IDL-B. In contrast, HDL-C, TG, glucose, ALT after correction for multiple comparisons, anthropometric measurements, and the investigated surrogate indices of adiposity and metabolic dysfunction did not show statistically significant changes. The absence of significant changes in these surrogate indices should not be interpreted as evidence that insulin resistance, metabolic syndrome, or MASLD was absent or unaffected. Rather, the findings indicate that no measurable change was detected in these indirect markers during the study period.

The metabolic indices may suggest a tendency toward preferential accumulation of adipose tissue in the visceral region and redistribution of body fat. However, based on these surrogate indices, the menopausal women in our study did not exhibit values consistent with insulin resistance, metabolic syndrome, or MASLD. This metabolic pattern may be associated with oestrogen deficiency during menopause. Nevertheless, most indices were close to borderline or at-risk thresholds, highlighting the importance of timely lifestyle and nutritional interventions aimed at improving metabolic health and preventing further disease progression.

Among the fundamental measures for improving metabolic health are dietary modifications, including reducing the intake of processed foods, salt, and foods with a high glycaemic index. Emphasis should be placed on the consumption of healthy fats and an adequate intake of protein, which may help protect against the age-related loss of skeletal muscle mass. Owing to its satiating effect, sufficient protein intake may also reduce overall food consumption, thereby contributing to body weight management. Furthermore, the inclusion of dietary sources of calcium, vitamin D, and phytoestrogens may be beneficial, as these nutrients may help alleviate some menopausal symptoms. Establishing regular daily routines related to eating habits, physical activity, and sleep is also recommended.

The findings of this exploratory intervention study indicate that regular consumption of ground flaxseed was associated with changes in selected lipid parameters and cholesterol subfractions in menopausal women. Given the exploratory nature of the study, these findings require confirmation in larger, adequately powered controlled trials.

We consider the inclusion of comprehensive intervention programmes for menopausal women to be appropriate. The study by Simkin-Silverman et al. [85] provides an example of such a comprehensive approach to addressing the consequences of menopause. Their study, which involved women aged 44–50 years, demonstrated that lifestyle modification has beneficial effects on body composition. The intervention programme comprised educational sessions focused on nutrition, cooking, and physical activity, together with ongoing support and motivational strategies to promote adherence to the prescribed lifestyle changes. Sustained weight maintenance or weight loss was also associated with improvements in LDL-C, triglyceride levels, and systolic blood pressure. Furthermore, women in the intervention group showed greater adherence to the physical activity targets and reported lower total energy intake than those in the control group. These findings suggest that comprehensive lifestyle interventions and educational programmes may effectively attenuate unfavourable changes in body composition during the transition from perimenopause to postmenopause.

### Study Limitations

Several study limitations should be considered when interpreting the results. Although the daily intake of 40 g of ground flaxseed used in this study was comparable to doses used in previous clinical studies, the intervention lasted only eight weeks. The relatively short intervention period, together with the relatively small number of female participants (n = 34), the lack of a comprehensive assessment of dietary habits, physical activity, and individual variability in metabolic responses, may have influenced the magnitude of the observed changes and the statistical power of the analyses. In addition, the study did not include a control group, and the investigated parameters were assessed using a pre–post design. Therefore, the observed changes cannot be definitively attributed to ground flaxseed consumption, as temporal variation, regression to the mean, or other uncontrolled factors may have contributed to the findings. Accordingly, the results should be considered exploratory and interpreted with caution. Larger, longer-term randomised controlled trials are needed to confirm and further investigate these findings.

## 5. Conclusions

Flaxseed supplementation was associated with a heterogeneous lipid response, characterized by a significant reduction in LDL-C alongside increases in total cholesterol and several triglyceride-rich lipoprotein fractions, including VLDL and IDL. Thus, the observed changes do not indicate a uniformly favorable lipid response, and their underlying mechanisms require further investigation. No statistically significant changes were observed in HDL-C, triglycerides, glucose, anthropometric parameters, or surrogate indices of adiposity and metabolic dysfunction after correction for multiple comparisons. The lack of significant changes in these surrogate indices should not be interpreted as evidence that insulin resistance, metabolic syndrome, or MASLD was absent or did not progress, but rather as an indication that no measurable changes in these indirect markers occurred during the study period.

The observed metabolic profile in menopausal women may suggest a tendency toward visceral adipose tissue accumulation and altered body-fat distribution, which may be partly related to the metabolic effects associated with oestrogen deficiency. Although the investigated indices did not indicate values consistent with insulin resistance, metabolic syndrome, or MASLD, their proximity to borderline or at-risk thresholds highlights the importance of early preventive strategies. Overall, these findings suggest a potential role for flaxseed supplementation as part of a broader lifestyle and nutritional approach aimed at improving cardiometabolic health in menopausal women and reducing the risk of future metabolic deterioration.

## Figures and Tables

**Table 1:** Nutritional and bioactive composition of the flaxseeds used in the intervention.

Parameter	Value	Parameter	Value
Dry matter	936.72 g/kg	Linoleic Acid	33.54%
Crude protein	192.33 g/kg	α-Linolenic Acid	38.08%
Crude fat	321.61 g/kg	Oleic Acid	16.87%
Ash	63.73 g/kg	Palmitic Acid	5.82%
Crude fibre	119.77 g/kg	Stearic Acid	4.12%
Sugars	16.01 g/kg	PUFA	71.62%
Lignans-SECO	5272.60 mg/kg	MUFA	16.87%
C. glycosides	2507.75 mg/kg	SFA	10.37%

C. glycosides—cyanogenic glycosides; SECO—secoisolariciresinol; PUFA—polyunsaturated fatty acid; MUFA—monounsaturated fatty acid; SFA—saturated fatty acid.

**Table 2:** Equations for calculating metabolic dysfunction indices [40].

Index	Equation
BMI (kg/m^2^)	BMI = weight (kg)/height^2^ (m)
WHR	WHR = WC (cm)/hip circumference (cm)
WHtR	WHtR = WC (cm)/height (cm)
BRI	BRI = 364.2 − 365.5 [1 − π^−2^ × WC^2^ (m) × height ^−2^ (m)]^1/2^
C-index	C-index = 0.109^−1^ × WC (m) × [weight (kg)/height (m)]^−1/2^
HSI	HSI = 8 × ALT/AST + BMI (+2, if diabetes or female)
LAP	LAP = [WC (cm) − a] × TG (mmol/L) (a = 65 for male; 58 for female)
TyG index	TyG index = Ln [TG (mg/dL) × FPG (mg/dL)/2]
TyG-BMI	TyG-BMI = TyG × BMI
TyG-WC	TyG-WC = TyG × WC
TyG-WHtR	TyG-WHtR = TyG × WHtR

BMI—body mass index; WHR—waist to hip ratio; WHtR—waist to height ratio; BRI—body roundness index; C-index—conicity index; HSI—hepatic steatosis index; LAP—lipid accumulation product; TyG-I—triglyceride–glucose index; TyG-BMI—triglyceride–glucose–body mass index; TyG-WC—triglyceride–glucose–waist circumference; TyG-WHtR—triglyceride–glucose–waist-to-height ratio.

**Table 3:** Baseline characteristics of probands.

Parameter	Med	IQR	Min	Max
Age (years)	48.50	47.00–52.50	45.00	55.00
Height (m)	1.68	1.64–1.72	1.58	1.78
Weight (kg)	69.90	62.60–83.50	53.50	114.00
BMI (kg/m^2^)	24.35	22.50–30.60	17.50	39.50
WHR	0.93	0.89–0.99	0.82	1.08
WHtR	0.54	0.48–0.63	0.41	0.76
WC (cm)	89.75	83.80–102.80	72.50	128.50
HC (cm)	96.25	93.57–104.52	87.30	118.80
FFM (kg)	47.60	45.00–50.48	40.70	58.70
MM (kg)	26.10	24.80–27.90	21.90	32.70
BFM (kg)	22.45	17.93–31.73	9.50	55.50
BFM (%)	33.30	27.15–39.25	17.10	48.60
VFA (cm^2^)	96.55	77.85–126.88	40.00	189.80
BMR (kcal)	1398.50	1342.25–1460.75	1250.00	1638.00
FBG (mmol/L)	4.77	4.54–5.22	4.06	6.53
T-C (mmol/L)	5.51	4.98–6.27	3.65	7.80
LDL-C (mmol/L)	3.34	2.74–3.89	1.70	5.18
HDL-C (mmol/L)	1.47	1.31–1.78	0.98	2.19
TG (mmol/L)	0.88	0.72–1.39	0.39	5.00
ALT (μkat/L)	0.28	0.19–0.35	0.15	0.83
AST(μkat/L)	0.29	0.25–0.36	0.19	0.43

BMI—body mass index; WHR—waist to hip ratio; WHtR—waist to height ratio; WC—waist circumference; HC—hip circumference; FFM—fat-free mass; MM—muscle mass; BFM—body fat mass; VFA—visceral fat area; BMR—basal metabolic rate; FBG—fasting blood glucose; T-C—total cholesterol; LDL-C—LDL cholesterol; HDL-C—HDL cholesterol; TG—triglycerides; ALT—alanine aminotransferase; AST—aspartate aminotransferase. Data are presented as median (Med) and interquartile range (IQR); Min—minimum; Max—maximum.

**Table 4:** Changes in selected biochemical parameters before and after intervention.

Parameter		Med (IQR)	Min	Max	W	Z	*p*/Holm *p*	r_r_b	95% CI
T-C (mmol/L)	before	5.51 (4.98–6.27)	3.65	7.80	80.00	4.17	˂0.001/˂0.001	0.822	[0.708, 1.000]
	after	6.18 (5.58–6.78)	4.07	9.50					
LDL-C (mmol/L)	before	3.34 (2.74–3.89)	1.70	5.18	12.00	−4.88	˂0.001/˂0.001	−0.960	[−0.997, −0.866]
	after	2.89 (2.28–3.18)	1.42	4.65					
HDL-C (mmol/L)	before	1.47 (1.31–1.78)	0.98	2.19	282.00	−1.47	0.142/1.000	−0.297	[−0.612, 0.145]
	after	1.51 (1.26–1.73)	0.72	2.09					
TG (mmol/L)	before	0.88 (0.72–1.39)	0.39	5.00	243.00	1.17	0.242/0.3619	0.234	[−0.155, 0.601]
	after	0.97 (0.72–1.40)	0.46	4.60					
FBG (mmol/L)	before	4.77 (4.54–5.22)	4.06	6.53	181.00	−1.55	0.120/0.3619	−0.314	[−0.659, 0.080]
	after	4.63 (4.45–4.98)	3.68	6.43					
ALT (µkat/L)	before	0.28 (0.19–0.35)	0.15	0.83	142.00	−2.08	0.037/0.1501	−0.427	[−0.760, −0.046]
	after	0.24 (0.19–0.33)	0.13	0.61					
AST (µkat/L)	before	0.29 (0.25–0.36)	0.19	0.43	0.95	−2.83	0.004/0.023	−0.591	[−0.886, −0.239]
	after	0.28 (0.24–0.34)	0.19	0.41					

T-C—total cholesterol; LDL—LDL cholesterol; HDL-C—HDL cholesterol; TG—triacylglycerols; FBG—fasting blood glucose; ALT—alanine aminotransferase; AST—aspartate aminotransferase. Data are presented as median and interquartile range, Med (IQR); minimum–maximum, Min–Max; Wilcoxon test statistic, W; standardized test statistic, Z; *p*-value/Holm-adjusted *p, p*/Holm *p*; Rank-biserial correlation, r_r_b; 95% confidence interval, 95% CI.

**Table 5:** Changes in cholesterol subfractions before and after intervention.

Parameter		Med	Min–Max	W	Z	*p*/Holm *p*	r_r_b	95% CI
VLDL (mmol/L)	Before	1.25 (1.11–1.49)	0.67–2.14	69.5	3.77	˂0.001/0.005	0.752	[0.471, 0.950]
	After	1.55 (1.18–1.65)	0.85–4.37					
IDL-C (mmol/L)	Before	0.46 (0.41–0.62)	0.20–1.03	247.00	1.08	0.282/1.000	0.218	[−0.005, 0.722]
	After	0.50 (0.41–0.59)	0.18–1.62					
IDL-B (mmol/L)	Before	0.34 (0.25–0.38)	0.15–0.64	92.00	3.48	˂0.001/0.010	0.708	[0.619, 0.943]
	After	0.41 (0.31–0.56)	0.15–0.90					
IDL-A (mmol/L)	Before	0.46 (0.36–0.64)	0.31–0.93	78.00	4.17	˂0.001/˂0.001	0.820	[0.733, 1.000]
	After	0.68 (0.54–0.87)	0.12–1.31					
LDL-1 (mmol/L)	Before	0.84 (0.72–1.08)	0.49–1.29	248.00	0.97	0.166/1.000	0.272	[−0.076, 0.690]
	After	0.90 (0.74–1.18)	0.15–1.39					
LDL-2 (mmol/L)	Before	0.37 (0.28–0.54)	0.12–0.82	188.5	−1.17	0.243/1.000	−0.240	[−0.390, 0.205]
	After	0.28 (0.23–0.54)	0.05–1.03					
LDL-3–7 (mmol/L)	Before	0.05 (0.00–0.15)	0.00–1.08	288.00	−0.74	0.461/1.000	−0.165	[−0.434, 0.478]
	After	0.01 (0.00–0.12)	0.00–0.82					
HDL-C (mmol/L)	Before	1.47 (1.31–1.78)	0.98–2.19	282.00	−1.47	0.142/1.000	−0.297	[−0.612, 0.145]
	After	1.51 (1.26–1.73)	0.72–2.09					
m-LDL size (nm)	Before	27.10 (26.80–27.40)	25.60–27.50	231.00	1.53	0.127/1.000	0.318	[0.000, 0.758]
	after	27.30 (26.90–27.40)	24.80–27.60					

VLDL—very low-density lipoprotein; IDL—intermediate-density lipoprotein; LDL—low-density lipoprotein; HDL-C—high-density lipoprotein; m-LDL size—mean LDL particle size. Data are presented as median and interquartile range, Med (IQR); minimum–maximum, Min–Max; Wilcoxon test statistic, W; standardized test statistic, Z; *p*-value/Holm-adjusted *p, p*/Holm *p*; Rank-biserial correlation, r_r_b; 95% confidence interval, 95% CI.

**Table 6:** Changes in selected anthropometric parameters and metabolic indices before and after intervention.

Parameter		Med (IQR)	Min–Max	W	Z	*p*/Holm *p*	r_r_b	95% CI
	after	24.50 (22.40–30.60)	17.60–39.50					
WHR	before	0.93 (0.89–0.99)	0.82–1.08	238.00	1.26	0.206/1.000	0.290	[−0.225, 0.704]
	after	0.93 (0.89–1.01)	0.82–1.10					
WHtR	before	0.54 (0.48–0.63)	0.41–0.76	75.50	1.14	0.251/1.000	0.281	[−0.200, 0.702]
	after	0.54 (0.48–0.64)	0.41–0.77					
WC (cm)	before	89.75 (83.80–102.80)	72.50–128.50	229.00	1.20	0.231/1.000	0.239	[−0.224, 0.662]
	after	89.75 (84.10–105.80)	72.20–130.50					
HC (cm)	before	96.25 (93.57–104.52)	87.30–118.80	177.00	−1.62	0.105/1.000	−0.333	[−0.714, 0.256]
	after	96.55 (93.40–104.70)	87.60–118.30					
VFA (cm^2^)	before	96.55 (77.85–126.88)	40.00–189.80	281.00	−0.32	0.745/1.000	−0.064	[−0.593, 0.212]
	after	92.95 (78.30–133.05)	41.90–194.40					
C-I	before	1.27(1.24–1.35)	1.15–1.44	128.00	1.492	0.136/1.000	0.323	[−0.108, 0.677]
	after	1.29(1.24–1.36)	1.15–1.46					
BRI	before	4.14 (3.12–6.24)	1.85–9.44	219.5	1.09	0.276/1.000	0.217	[−0.185, 0.572]
	after	4.23 (3.05–6.36)	1.83–9.79					
HSI	before	33.16 (30.83–40.38)	24.50–53.69	234.0	−1.08	0.278/1.000	−0.213	[−0.573, 0.176]
	after	33.25 (31.31–39.55)	25.16–51.24					
LAP	before	30.40 (16.29–46.25)	7.33–52.50	199.00	1.68	0.092/1.000	0.331	[−0.059, 0.667]
	after	31.11 (15.27–68.49)	8.66–74.28					
TyG-I	before	8.08 (7.86–8.60)	7.35–10.01	229.00	1.17	0.241/1.000	0.230	[−0.168, 0.582]
	after	8.20 (7.85–8.56)	7.35–11.07					
TyG-BMI	before	199.84 (176.31–248.85)	137.02–395.59	237.0	1.03	0.301/1.000	0.203	[−0.180, 0.563]
	after	199.66 (173.00–263.92)	135.16–394.15					
TyG-WC	before	729.41 (646.98–861.30)	565.00–1286.92	201.0	1.65	0.099/1.000	0.324	[−0.049, 0.657]
	after	726.01 (654.91–932.25)	554.46–1302.21					
TyG-WHtR	before	4.40 (3.91–5.16)	3.24–7.61	192.5	1.57	0.116/1.000	0.314	[−0.067, 0.652]
	after	4.40 (3.96–5.45)	3.17–7.71					

WHR—waist to hip ratio; WHtR—waist to height ratio; WC—waist circumference; HC—hip circumference; VFA—visceral fat area; C-I—conicity index; BRI—body roundness index; HSI—hepatic steatosis index; LAP—lipid accumulation product; TyG-I—triglyceride–glucose index; TyG-BMI—triglyceride–glucose–body mass index; TyG-WC—triglyceride–glucose–waist circumference; TyG-WHtR—triglyceride–glucose–waist-to-height ratio. Data are presented as median and interquartile range, Med (IQR); minimum–maximum, Min–Max; Wilcoxon test statistic, W; standardized test statistic, Z; *p*-value/Holm-adjusted *p, p*/Holm *p*; Rank-biserial correlation, r_r_b; 95% confidence interval, 95% CI.

**Table 7:** Spearman’s rank correlation coefficients between selected anthropometric parameters, metabolic indices, and lipoprotein subfractions based on post-intervention values (n = 34).

Parameter	TyG-I	TyG-BMI	TyG-WC	TyG-WHtR
BMI	0.714 ***	0.956 *** ^M^	0.933 ***	0.939 ***
WC	0.649 ***	0.909 ***	0.947 *** ^M^	0.915 *** ^M^
VFA	0.570 ***	0.860 ***	0.900 ***	0.864 ***
WHtR	0.673 ***	0.922 ***	0.936 *** ^M^	0.937 *** ^M^
LAP	0.922 ***	0.971 ***	0.971 *** ^M^	0.974 *** ^M^
HSI	0.739 ***	0.899 *** ^M^	0.834 ***	0.879 ***
FBG	0.499 ** ^M^	0.454 * ^M^	0.434 * ^M^	0.464 ** ^M^
ALT	0.592 ***	0.562 ***	0.495 **	0.537 **
TG	0.988 *** ^M^	0.858 *** ^M^	0.832 *** ^M^	0.861 *** ^M^
T-C	0.465 **	0.452 **	0.446 **	0.451 **
LDL-C	0.399 *	0.420 *	0.405 *	0.431 *
HDL-C	−0.413 *	−0.485 **	−0.483 **	−0.485 **
IDL-C	0.702 ***	0.712 ***	0.709 ***	0.724 ***
LDL-3–7	0.580 ***	0.447 **	0.446 **	0.458 **
m-LDL size	−0.587 ***	−0.441 **	−0.426 *	−0.443 **

Spearman’s rank correlation coefficients (ρ) are presented. No adjustment for multiple comparisons was applied. * *p* < 0.05; ** *p* < 0.01; *** *p* < 0.001. ^M^ Mathematically coupled variables. BMI—body mass index; WC—waist circumference; VFA—visceral fat area; WHtR—waist to height ratio; LAP—lipid accumulation product; HSI—hepatic steatosis index; FBG—fasting blood glucose; ALT—alanine aminotransferase; TG—triacylglycerols; T-C—total cholesterol; LDL-C—LDL cholesterol; HDL-C—HDL cholesterol; IDL-C—intermediate-density lipoprotein; m-LDL size—mean LDL particle size.

## Data Availability

The data presented in this study are available on request from the corresponding author due to privacy.

## Abbreviations

The following abbreviations are used in this manuscript:

ALT	Alanine aminotransferase
AST	Aspartate aminotransferase
BFM	Body fat mass
BMI	Body mass index
BMR	Basal metabolic rate
BRI	Body roundness index
C-I	Conicity index
FBG	Fasting blood glucose
FFM	Fat-free mass
HC	Hip circumference
HDL	High-density lipoprotein
HSI	Hepatic steatosis index
IDL-A	Intermediate-density lipoprotein-A
IDL-B	Intermediate-density lipoprotein-B
IDL-C	Intermediate-density lipoprotein-C
IQR	Interquartile range
LAP	Lipid accumulation product
LDL	Low-density lipoprotein
MM	Muscle mass
MUFA	Monounsaturated fatty acid
PUFA	Polyunsaturated fatty acid
SECO	Secoisolariciresinol
SDG	Secoisolariciresinol diglucoside
SFA	Saturated fatty acid
T-C	Total cholesterol
TG	Triglyceride
TyG-I	Triglyceride–glucose index
TyG-BMI	Triglyceride–glucose index–BMI
TyG-WC	Triglyceride–glucose index–WC
TyG-WHtR	Triglyceride–glucose index–WHtR
VFA	Visceral fat area
VLDL	Very low-density lipoprotein
WC	Waist circumference
WHR	Waist to hip ratio
WHtR	Waist to height ratio
